# Initial Clinical Experience of a Novel Shapeable Bolus for Radiotherapy in a Patient With a Facial Cutaneous Squamous Cell Carcinoma: A Case Report

**DOI:** 10.7759/cureus.57415

**Published:** 2024-04-01

**Authors:** Kazuki Wakabayashi, Hajime Monzen, Hiroshi Doi, Takaya Inagaki, Tetsuo Sonomura

**Affiliations:** 1 Department of Central Radiology, Wakayama Medical University Hospital, Wakayama, JPN; 2 Department of Medical Physics, Graduate School of Medical Sciences, Kindai University, Osakasayama, JPN; 3 Department of Radiation Oncology, Faculty of Medicine, Kindai University, Osakasayama, JPN; 4 Department of Radiology, Wakayama Medical University, Wakayama, JPN

**Keywords:** shapeable, skin cancers, radiation therapy, bolus, cutaneous squamous cell carcinoma (scc)

## Abstract

Radiation therapy with X-rays for skin cancer uses a bolus to increase the surface dose. Commercial gel sheet boluses adhere poorly to the patient’s body because of surface irregularities. This causes an air gap and reduces the surface dose. We have developed a novel shapeable bolus (HM bolus; Hayakawa Rubber Co., Ltd., Hiroshima, Japan), and we describe the first clinical application of this bolus here. The case was an 82-year-old male with a facial cutaneous squamous cell carcinoma. The postoperative radiotherapy plan using the HM bolus provided a more uniform dose to the target compared with a plan without the HM bolus. The HM bolus adhered stably to the patient’s skin, and there were no issues with its clinical use.

## Introduction

Skin cancer, including cutaneous squamous cell carcinoma (cSCC), accounts for 2.5% of all cancers in Japan [1] and 20%‒30% of nonmelanoma skin cancers [2]. The standard treatment for spinous cell carcinoma is surgery. Alternatively, radiotherapy may be considered if surgery is not desirable for functional or cosmetic reasons, such as in cases involving the face (eyelids, nose, or lips) or fingers. Radiotherapy is also indicated if the tumor is too large, sufficient margins cannot be secured, there are multiple lesions, postoperative recurrence has been reported, surgery is difficult for medical reasons, or surgery is rejected [3]. Indications for postoperative irradiation include nonradical surgery, such as positive or close resection margins, perineural invasion, multiple lymph node metastases, extracapsular invasion, intracranial invasion, chronic immunodeficiency, recurrence, and tumors with a high risk of recurrences, such as T3 and T4 cSCC [3]. A systematic review of cSCC reported local recurrence after external radiotherapy with a pooled average local recurrence rate of 6.4% [4].

Megavoltage (MV) X-rays and electron beams used in radiotherapy have a buildup effect, which reduces the dose administered to the patient’s skin surface. Therefore, a bolus is used to increase the dose that reaches the skin surface [5]. A commercial gel-sheet bolus is commonly used for this purpose. However, a gel-sheet bolus does not adhere well to irregular body surfaces, which causes unexpected air gaps between the skin and the bolus, reducing the surface dose [6]. For example, in the volumetric modulated arc therapy technique, a 10 mm air gap reduced the surface dose by an average of 13.6% [7]. The ideal bolus should adhere closely to the patient’s skin without air gaps. The HM bolus (Hayakawa Rubber Co., Ltd., Hiroshima, Japan) was recently developed by Wakabayashi et al. and Nakamura et al. [8,9]. The HM bolus has unique features, including tissue equivalence, transparency, and reusability, and it can be easily shaped by hand after warming it to approximately 40 °C, allowing it to adhere closely to the skin. We aim to evaluate the feasibility of using this bolus in clinical settings. In this paper, we report the first clinical application of the HM bolus worldwide for the treatment of cSCC.

## Case presentation

**Figure 1:**
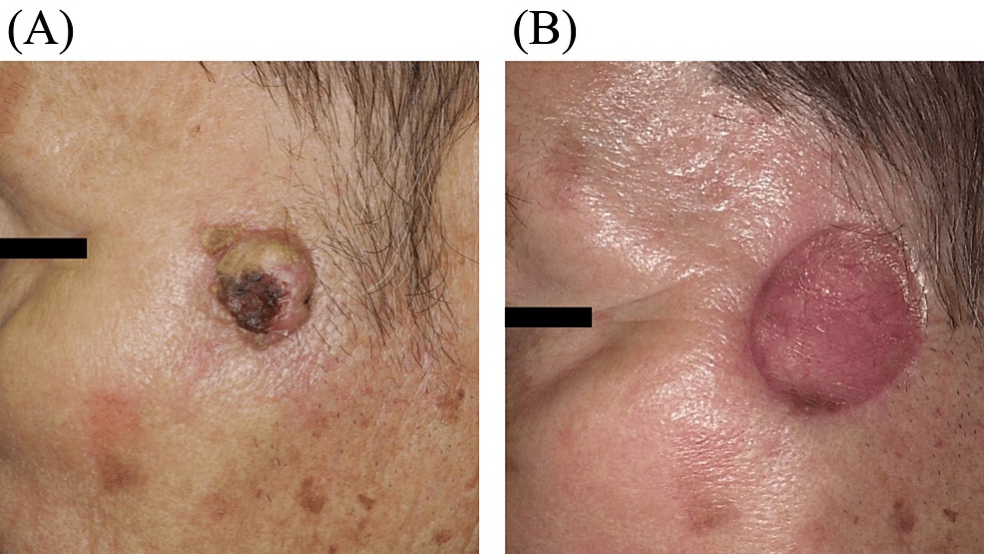
(A) Preoperative and (B) postoperative photographs of the facial lesion.

An 82-year-old man had been aware of a tumor on his left cheek since 2022 (Figure 1A). The tumor was pathologically diagnosed as cSCC following a biopsy (May 2023). Ultrasonography revealed a tumor with a diameter of 17 mm (June 2023). Fluoro-2-deoxyglucose-enhanced positron emission tomography combined with CT confirmed the absence of metastases. He underwent surgery (July 2023), which involved tumor excision and a full-thickness skin graft (Figure 1B). Postoperative pathology showed positive margins, and the patient was referred to our department for postoperative radiotherapy (September 2023). The total prescribed radiation dose was 5500 centigray (cGy) in 20 fractions. The local ethics committee approved the use of the HM bolus in this patient (approval no. 4052), and the patient provided written informed consent for the publication of this case.

**Figure 2:**
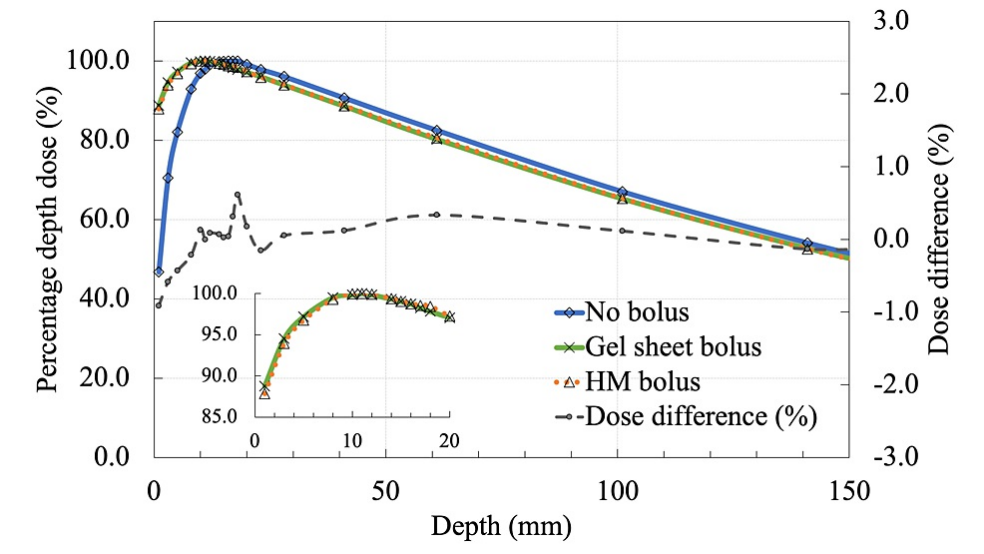
Percentage depth doses and dose differences between the HM bolus and gel-sheet bolus for a 6 MV photon beam.

The HM bolus was used because the target lesion was in an irregular cheek area, and a commercial gel-sheet bolus was unlikely to adhere well to the skin, resulting in an air gap. Before clinical use, the dose characteristics of the HM bolus were evaluated. The HM bolus was placed on a water-equivalent phantom (Tough Water Phantom; Kyoto Kagaku Co., Ltd., Kyoto, Japan), and the percentage depth doses (PDDs) of a 6 MV photon beam (Synergy; Elekta AB, Stockholm, Sweden) were determined using a parallel-plate ionization chamber (Roos Type 34001; PTW, Freiburg, Germany) and a smart electrometer (RAMTEC; Toyo Medic, Tokyo, Japan). The PDDs of the HM bolus were compared with those of a commercially available gel-sheet bolus (Bolx-I; CIVCO Medical Solution, Orange City, IA). The thickness of the HM bolus and gel-sheet bolus was 5 mm. Experiments were performed using the same geometry as reported by Nakamura et al. [9]. The PDD results are shown in Figure 2, which indicate that the HM bolus agreed with the gel-sheet bolus within 1% of the dose difference.

**Figure 3:**
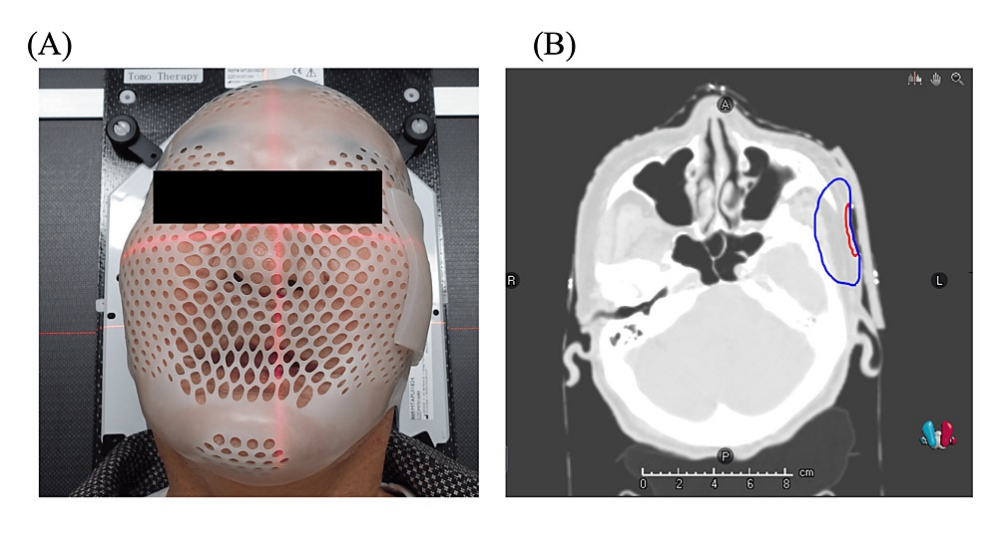
Radiotherapy setup using the HM bolus. (A) The HM bolus adhered closely to the facial irregularities. (B) Axial computed tomography image. The red line indicates the tumor bed and the blue line indicates the planning target volume. The airgap, the distance between the skin and the HM bolus, was 2.7 mm.

The patient’s head was immobilized using a thermoplastic mask (CIVCO Medical Solution) to undergo a computed tomography (CT) scan for radiotherapy planning. The HM bolus was heated in a hot water bath (UW-35; Taiji Co., Ltd., Kanagawa, Japan) (70 °C) for approximately 30 s to warm its surface temperature to approximately 40‒43 °C, and it was then shaped onto the thermoplastic mask. The HM bolus and patient setup are shown in Figure 3A. The air gap between the HM bolus and the patient’s skin surface, measured using RayStation (RaySearch Laboratories AB, Stockholm, Sweden) on a CT image, was consistently <3 mm. The air gap was considered to be due to inadequate adhesion between the thermoplastic mask and the skin surface. The HM bolus was only heated to 40‒43 °C, whereas the thermoplastic mask was heated to approximately 70°C. We required no additional time to allow the HM bolus to cool, and the patient did not suffer any burns from the HM bolus.

**Figure 4:**
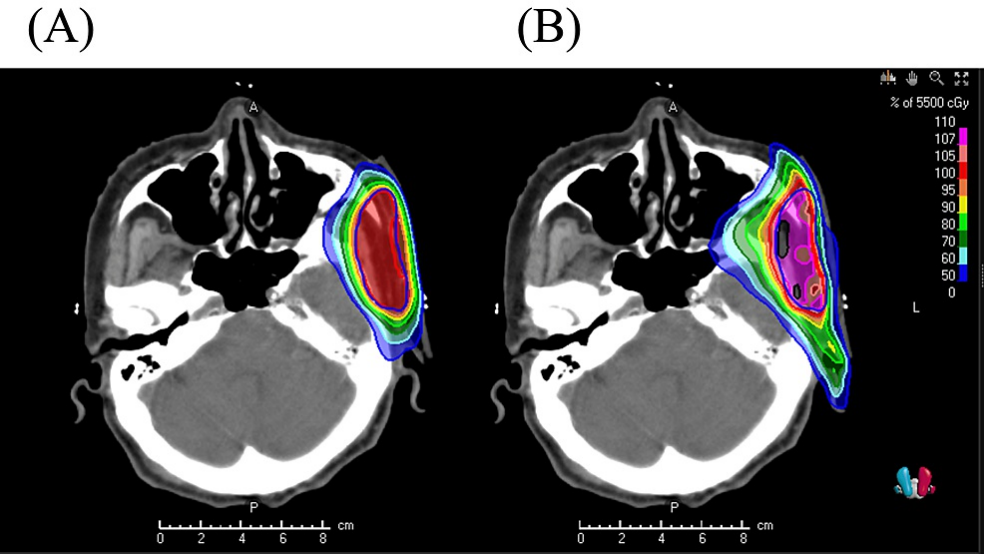
Isodose lines for the cutaneous squamous cell carcinoma using volumetric modulated arc therapy (A) with and (B) without the HM bolus.

**Figure 5:**
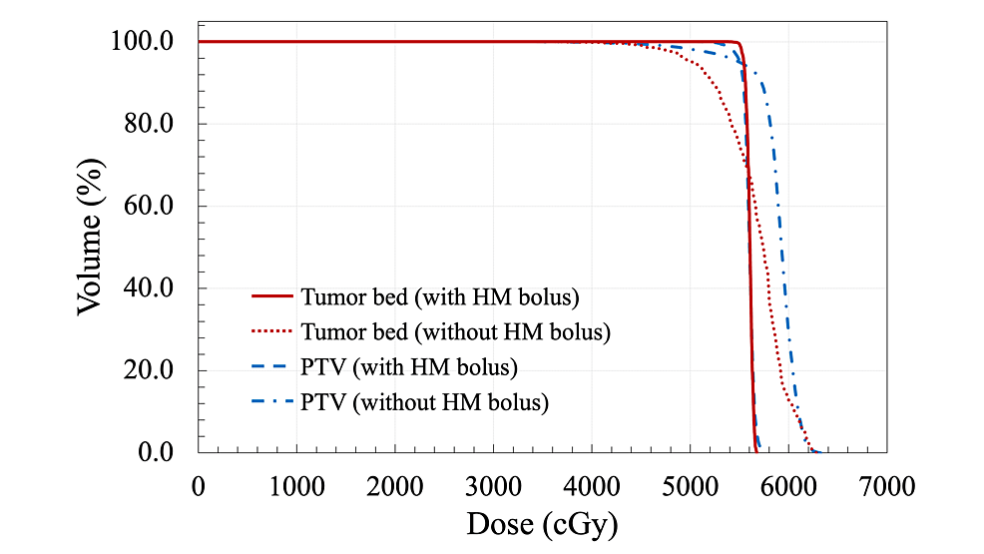
Dose-volume histogram for the radiotherapy plans with (solid lines) or without (dotted lines) the HM bolus. The red lines indicate the tumor bed and the blue lines indicate the planning target volume (PTV).

CT images were obtained using an Aquilion LB CT system (Canon Medical Systems, Tochigi, Japan) (Figure 3B). The imaging conditions were as follows: tube voltage = 120 kV, tube current = 300 mA, matrix size = 512 × 512 pixels, field of view = 500 mm, and slice thickness = 2 mm. The CT images were transferred to the RayStation, and the target and risk organs were contoured. The tumor bed + 10 mm was defined as the clinical target volume (CTV). The planning target volume (PTV) was defined as a 5 mm expansion from the CTV. The treatment technique of choice was two-arc (0°‒179°) volumetric modulated arc therapy with 6 MV X-rays. Treatment was optimized to ensure that the prescribed dose was administered to 95% of the PTV. For comparison, a separate plan was prepared by replacing the HM bolus with air density (without the HM bolus plan). The dose was calculated using the collapsed cone convolution algorithm with RayStation. The following dosimetric parameters were determined for each bolus condition: D98% (dose covering 98% of the PTV; near minimum dose), D50% (median dose), D2% (near maximum dose), and conformity number, CN, which was defined as follows [10]:

$\large CN=\frac{TV_{RI}}{TV}\times \frac{TV_{RI}}{V_{RI}}$

where TVRI is the target volume covered by the reference isodose, TV is the target volume, and VRI is the volume of the reference isodose. The isodose lines, dose-volume histogram, and dosimetric parameters of each bolus plan are shown in Figures 4-5 and Table 1. In the plan with the HM bolus, the prescription dose covered the PTV uniformly with no hotspots exceeding 107%. By contrast, in the plan without the HM bolus, the prescribed dose achieved 95% coverage of the PTV; however, there was a decrease in the dose that would reach the tumor bed.

**Table 1:** Dosimetric parameters of the radiotherapy plans with and without the HM bolus. The radiotherapy plan was optimized to ensure that the prescribed dose was administered to 95% of the PTV. CN, conformity number; D98%, dose covering 98% of the PTV (near minimum dose), D50%, median dose; D2%, near maximum dose; PTV, planning target volume

		With HM Bolus	Without HM Bolus
Tumor bed	D_98%_ (cGy)	5,520	4,738
	D_50% _(cGy)	5,607	5,742
	D_2% _(cGy)	5,660	6,211
PTV	D_98%_ (cGy)	5,426	5,054
	D_50% _(cGy)	5,600	5,925
	D_2% _(cGy)	5,698	6,190
CN		0.90	0.79

The planned postoperative radiotherapy was successfully completed. In terms of toxicities, which were classified using the Common Terminology Criteria for Adverse Events version 5.0 [11], grade 1 dermatitis was observed at the end of treatment. There were no toxicities exceeding grade 1. The patient did not experience any recurrence at one month after the completion of radiotherapy.

## Discussion

We have developed a shapeable soft-rubber bolus and reported its adherence to a head-phantom [8]. Nakamura et al. (2023) reported an HM bolus with improved temperature characteristics and transparency, which were limitations of the existing soft-rubber boluses [9]. This report describes the first clinical use of the HM bolus in a patient with a cSCC of the cheek. The face is a difficult area for a bolus to adhere to the skin because of its irregular contours. Therefore, several bolus studies have been published [8,9,12,13]. For instance, Park et al. reported that a bolus could be fabricated using a three-dimensional (3D) printer [14]. However, the 3D-printed bolus was hard, and the patient reported pain. The acrylonitrile butadiene styrene and polylactic acid resins commonly used for 3D-printed boluses have Shore hardnesses of 70D and 75D, respectively, which may cause discomfort to the patient’s sensitive skin [15]. The HM bolus was designed to allow shaping without the need for strong pressure at temperatures above 40°C [9]. It is important to be able to shape the bolus at lower temperatures to avoid low-temperature burns, which may be caused when temperatures above 44°C are needed to shape the bolus [16]. Furthermore, the HM bolus does not require a long fabrication time, unlike a 3D-printed bolus [17], and it can be easily shaped in the clinical setting, allowing timely treatment.

The HM bolus achieved a more uniform dose to the tumor bed and PTV compared to the plan prepared without the HM bolus. The HM bolus adhered very well to the shell, which may have avoided a reduction in the surface dose. The International Commission on Radiation Units and Measurements Report No. 83 recommends that the maximum dose applied to the target should be within 107% of the planned dose [18]. However, in the plan without the HM bolus, it was impossible to maintain the dose to both the PTV and surface.

Setting up a patient by using a surface guide system for surface-guided radiotherapy (SGRT) has been reported [19,20]. The HM bolus is transparent, allowing the clinical team to see the superficial targets [9], although there is the disadvantage that the HM bolus does not provide accurate positioning when using SGRT. In such cases, we recommend using a nontransparent soft-rubber bolus [8].

This case report is limited by the inclusion of a single case and the short-term follow-up. Therefore, we need a prospective randomized study with a larger sample size and longer-term follow-up data to show a broader generalization of the application of HM bolus.

## Conclusions

We present the first case report of postoperative radiotherapy using our novel HM bolus for a cSCC on a cheek. The bolus achieved accurate dose delivery during the treatment of this superficial tumor on an irregular skin surface. Moreover, the bolus can be easily shaped in the clinical setting, and it did not cause any low-temperature burns to the patient. This bolus offers considerable benefits for patients.
